# Research on Noise Reduction and Analysis of Reciprocating Friction Vibration Signals Based on the Complementary Ensemble Empirical Mode Decomposition

**DOI:** 10.3390/s26082433

**Published:** 2026-04-15

**Authors:** Yier Yu, Haijun Wei, Zongxiao Liu

**Affiliations:** Merchant Marine College, Shanghai Maritime University, Shanghai 201306, China; 13375753359@163.com (Y.Y.);

**Keywords:** frictional vibration signal, noise reduction processing, adaptive correlation coefficient, complementary ensemble empirical mode decomposition (CEEMD), multi-scale permutation entropy (MPE)

## Abstract

This paper presents an adaptive noise reduction method based on Complementary Ensemble Empirical Mode Decomposition (CEEMD) to address the non-stationary characteristics and noise interference present in friction vibration signals from mechanical equipment. and friction testing machine simulation experiments. The performance of CEEMD and Ensemble Empirical Mode Decomposition (EEMD) was compared through MATLAB R2023b simulations and experiments conducted on a friction testing machine. CEEMD achieved a computational efficiency 85.6% higher than that of EEMD and effectively reduced mode aliasing. Among them, the adaptive correlation coefficient screening method performed well in signal reconstruction, and the high correlation (correlation coefficient > 0.8) between the denoised signal and the laboratory noise signal was verified using the multi-scale permutation entropy (MPE) theory, which is of great significance for early diagnosis of mechanical faults, prediction of equipment life and timely maintenance decisions.

## 1. Introduction

Friction and wear are among the main causes of mechanical component failure, accounting for 50–70% of all failures of mechanical equipment. Acoustic emission (AE) technology plays a crucial role in equipment monitoring [1,2]. In marine shipping, reciprocating friction vibration signals from key friction pairs in the main engine (such as piston ring–cylinder liner) contain abundant equipment condition information. However, under the harsh working conditions of actual ships, such signals are easily submerged by environmental noise and multi-source vibrations. This poses significant challenges to vibration-based condition monitoring and early fault diagnosis. Therefore, developing effective noise reduction and analysis techniques for friction vibration signals is of great significance for ensuring marine navigation safety and implementing predictive maintenance.

To meet this demand, researchers worldwide have conducted extensive research on noise reduction and analysis of friction vibration signals. The Fourier transform, proposed by Fourier in 1807, is a fundamental tool for analyzing linear systems and stationary signals, but it lacks temporal localization and is unsuitable for non-stationary friction vibration signals. The short-time Fourier transform (STFT), introduced by Gabor in 1946, enables time–frequency analysis through a sliding window function. However, its fixed window length cannot simultaneously capture the high-frequency impacts and low-frequency trends present in reciprocating friction vibration signals, making it unsuitable for such non-stationary signals [3]. The Wigner-–Ville time–frequency distribution, developed in quantum mechanics, provides high-resolution signal analysis and allows real-time adjustment of time-frequency spectra. However, its joint time–frequency distribution is prone to cross-terms, which limits its applicability to complex friction signals [4]. The continuous wavelet transform proposed by J. Morlet in 1984, and the discrete wavelet transform introduced by Ingrid Daubechies in 1989, improved the analysis efficiency of time-varying signals. The subsequent wavelet packet transform further optimizes high-frequency resolution. Nevertheless, these methods are still limited by the predefinition of basis functions and show insufficient adaptability to nonlinear signals such as friction vibration [5].

In recent years, several advanced adaptive decomposition methods have been developed to address the limitations of traditional time-frequency techniques. For instance, the wavelet transform is computationally efficient but requires manual selection of basis functions, which limits its adaptability to non-stationary signals. Complete Ensemble Empirical Mode Decomposition with Adaptive Noise (CEEMDAN), a variant of EEMD, reduces residual noise at the cost of increased computational load. Variational Mode Decomposition (VMD) provides good frequency separation but requires the number of modes to be specified in advance, which is often unknown for real friction vibration signals. While these methods have proven effective in various applications, their performance on reciprocating friction vibration signals—which are characterized by strong non-stationarity and low signal-to-noise ratios—remains underexplored.

The limitations of the traditional time-frequency methods discussed above have spurred research into empirical mode decomposition (EMD) and its improved variants. Among them, Complementary Ensemble Empirical Mode Decomposition (CEEMD) has demonstrated application value in bearing fault diagnosis of unmanned aerial vehicles, bridge monitoring data denoising, and other fields. This is due to its advantages of adaptive decomposition and suppression of mode mixing [6,7].

Although Complementary Ensemble Empirical Mode Decomposition (CEEMD) has been successfully applied in bearing fault diagnosis and bridge monitoring, its potential for denoising reciprocating friction vibration signals in complex marine engine environments remains largely unexplored. To fill this gap, we propose an adaptive noise reduction method based on CEEMD, specifically designed for the cylinder liner–piston ring friction pair in marine main engines. The specific objectives are threefold: to compare CEEMD and EEMD in terms of decomposition efficiency and mode mixing suppression; to evaluate three intrinsic mode function (IMF) screening methods using signal-to-noise ratio (SNR), mean square error (MSE), and normalized cross-correlation (NCC) metrics; and to validate the denoising effectiveness under dry, boundary, and mixed friction conditions through multi-scale permutation entropy analysis.

## 2. Theoretical Basis and Algorithm

### 2.1. CEEMD Adaptive Correlation Coefficient Theory and Algorithm

CEEMD, like EEMD, is a noise-assisted method that uses a similar strategy to EEMD when extracting the first intrinsic mode function (IMF) [8,9]. In this approach, the operator *E_j_* (·) is defined as the j-th modality obtained by empirical mode decomposition (EMD) to a given signal. Here, *w^i^* represents Gaussian white noise with unit variance and zero mean, while the *ε_k_* controls the signal-to-noise ratio at each stage [10]. Let *x* denote the target signal. The execution steps of CEEMD are as follows.

First, the decomposition process is repeated *I* times using EMD with different noise realizations. The overall average is then calculated and defined as the first IMF of the target signal *x*.

(1) $${IMF}_{1}=\frac{1}{I}\sum_{i=1}^{I}E_{1}(x+\varepsilon_{0}w^{i})$$

Then, the first-order residual is calculated as

(2) $$r_{1}=x-{IMF}_{1}$$

Continue to decompose to achieve *r*_1_ + *ε*_1_*E*_1_(*w^i^*), where *i* = 1,…, *I*, until they satisfy their first IMF condition and define the overall mean as the second IMF:

(3) $${IMF}_{2}=\frac{1}{I}\sum_{i=1}^{I}{E_{1}(r}_{1}+\varepsilon_{1}E_{1}(w^{i}))$$

For *k* = 2,… *K*, calculate the k-order residual: *r_k_* = *r*_(*k*_−_1)_−*IMF_k_*, then extract the first IMF component of *r_k_* + *ε_k_E_k_*(*w^i^*), where *i* = 1,…, *I*, and calculate the overall mean of them to obtain the *IMF*_(*k*+1)_ of the target signal.

(4) $${IMF}_{\left(k+1\right)}=\frac{1}{I}\sum_{i=1}^{I}{E_{1}(r}_{k}+\varepsilon_{k}E_{k}(w^{i}))$$

Continue the screening process until the resulting residuals can no longer be decomposed (when the extremum of the residuals is no more than two at most); then, the result is obtained

(5) $$R=x-\sum_{k=1}^{K}{IMF}_{k}$$

where *R* is the final residual and *K* is the total number of IMFs. Therefore, the target signal *x* can be expressed as:

(6) $$x=\sum_{k=1}^{K}{IMF}_{k}+R$$

Implementing the CEEMD algorithm requires specifying several key parameters that directly affect decomposition quality, reproducibility, and the residual results discussed in Section 4.1. The parameters used in this study are summarized in Table 1, along with a brief justification for each choice.

### 2.2. Multi-Scale Permutation Entropy

Multi-scale permutation entropy (MPE) involves first constructing a coarse-grained version of the time series. This is done by dividing the original signal into multiple time windows (or scales) of different lengths and calculating the average value of the signal within each window to form a new time series. This approach allows the dynamic changes in the time series to be analyzed at different scales. For each coarse-grained time series, permutation entropy is applied to quantify its complexity or uncertainty. This involves dividing the time series into overlapping subsequences, calculating the permutation pattern of the elements in each subsequence, and then counting the frequency of occurrence of different patterns to obtain the entropy value. When applied to complex friction vibration signals, multi-scale permutation entropy helps remove noise and highlight key signal features.

Consider the time series {x(i), I = 1,2, N}, and coarse-graining it to obtain the coarse-graining sequence {yj (τ)}. The expression for yj (τ) is

(7) $$y_{j}^{\left(\tau \right)}=\frac{1}{\tau }\sum_{i=(j-1)\tau +1}^{j\tau }x_{i}\;\;j=1,2\cdots ,[\frac{N}{\tau }]$$

τ is the scale factor, τ = 1,2,…. Obviously τ = 1, the coarse-grained sequence is the original sequence. When τ > 1, the original sequence is coarse-grained to a coarse-grained sequence of length.

The permutation entropy value is calculated for each coarse-grained sequence, and the results are plotted as a function of the scale factor. This procedure is known as multi-scale permutation entropy analysis. Therefore, the scale factor should be chosen to be greater than 10 to ensure more effective smoothing of noise when analyzing time series. This helps highlight long-term trends and periodic patterns while improving the stability of statistical results. A larger scale factor helps to combine more data points, thereby reducing the impact of short-term fluctuations and enabling the analysis results to better reflect the essential characteristics and dynamic changes in the time series.

To reduce the influence of trend and seasonal components when calculating multi-scale permutation entropy for vibration signals under different friction states, the multi-scale partial mean parameter is introduced. The multi-scale permutation entropy curves for vibration signals under different friction conditions show distinct decreasing trends, indicating that the rate at which the complexity decreases varies under different friction states as the scale increases. Therefore, the partial mean of the multi-scale permutation entropy is used as an important characteristic indicator to distinguish these states.

The formula is as follows:

(8) $$H\;=\;(\frac{1+\left|\frac{3(m\;-\;c)}{d}\right|}{3})\cdot m$$

In the formula: m is the mean of the data set;

c is the median of the data set;

d is the standard deviation of the data set.

### 2.3. Theoretical Analysis of Three Friction States

The friction state of a friction pair is closely related to lubrication conditions, contact characteristics, and the relative motion state. The three typical friction states discussed in this paper—dry friction, boundary friction, and mixed friction—have distinct formation mechanisms and vibration response characteristics.

In the dry friction state, no lubricating oil exists between the contact surfaces. The two solid surfaces are in direct contact, and friction arises mainly from adhesion, plowing, and shear deformation of surface asperities. This state typically results in a high friction coefficient, strong impact, and high-amplitude vibration signals, accompanied by noticeable randomness and impulsiveness.

In the boundary friction state, a thin lubricating oil film is adsorbed onto the surfaces, but the film is too thin to completely separate the contacting asperities. Most of the load is still carried by the micro-asperities. Therefore, the vibration signal still contains obvious impact components, and the signal fluctuations are relatively significant.

In the mixed friction state, a continuous lubricating oil film forms between the contact surfaces. Part of the load is supported by the hydrodynamic pressure of the lubricating oil, while the remainder is borne by the contacting asperities. The friction coefficient is relatively low, the vibration tends to be stable, the amplitude is small, and the noise component is relatively weak.

The differences in friction characteristics and vibration signals under the three states provide a theoretical basis for the design of the experiment, the analysis of vibration signals, and the verification of the noise reduction method in this study.

## 3. Experimental Design and Data Acquisition

### 3.1. Experimental Framework Overview

**Figure 1:**
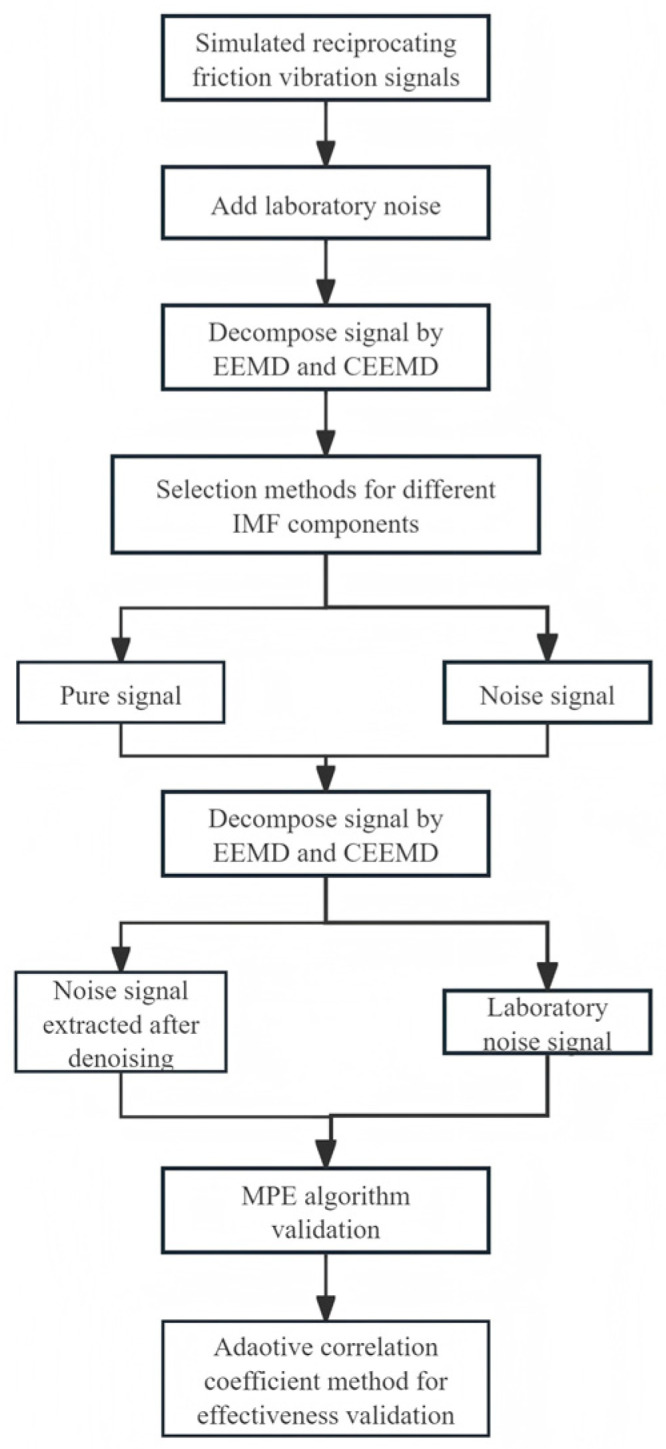
Overall experimental framework.

Figure 1 illustrates the overall framework of this study, providing a clear and systematic overview of the experimental methodology. The process begins with the construction of noisy simulated signals using MATLAB and noise collected from the laboratory. This is followed by comparative decomposition using EEMD and CEEMD. Three screening methods are then applied to select the optimal intrinsic mode functions (IMFs), with the adaptive correlation coefficient method achieving the best performance. On this basis, actual friction vibration signals are acquired using a BRUKER UMT tribometer under three typical lubrication conditions: mixed friction, boundary friction, and dry friction. The denoising effectiveness is subsequently validated through multi-scale permutation entropy (MPE) analysis, including MPE curve similarity comparison and partial mean calculation for friction state characterization.

### 3.2. Test Objects

To obtain reciprocating friction vibration signal data, we used a BRUKER UMT tribometer, a vibration signal analyzer, an acceleration sensor, and other equipment for signal simulation and acquisition. In the experiment, the pin specimen was fixed above the block specimen without contact, while the block specimen moved reciprocally at a preset frequency. In this state, since the friction pair has not contacted, the signal measured by the sensor is actually the noise signal of the laboratory.

A simulated pure signal was generated using MATLAB and combined with the laboratory noise signal. This allows us to study and compare the signals reconstructed by different screening methods after the CEEMD method. Then, friction vibration signals simulated by the testing machine under different states were used, combined with multi-scale permutation entropy and its partial mean, to evaluate the actual effectiveness of the noise reduction.

### 3.3. Test Equipment and Instruments as Well as Test Specimens

**Figure 2:**
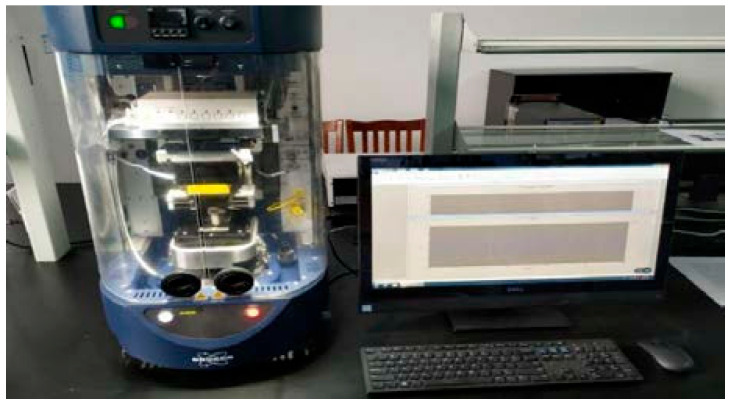
BRUKER UMT tribometer.

The BRUKER UMT friction and wear testing machine from Germany was used as the main equipment for the test, as shown in Figure 2. The equipment can adapt to various testing requirements and is mainly used for studying and evaluating the friction, wear and lubrication properties of materials and coatings.

**Figure 3:**
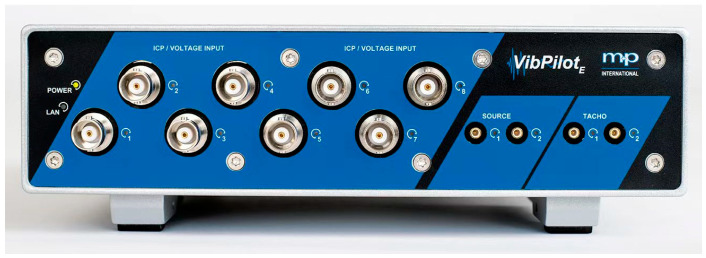
M-P type signal acquisition and analysis instrument.

The M+P signal acquisition analyzer was used to obtain the original vibration signals. The device can capture data at a high sampling rate, ensuring the acquisition of accurate vibration and noise signals, as shown in Figure 3. It also supports simultaneous data acquisition through multiple channels, allowing information to be received from multiple sensors and facilitating complex multi-point analysis.

**Figure 4:**
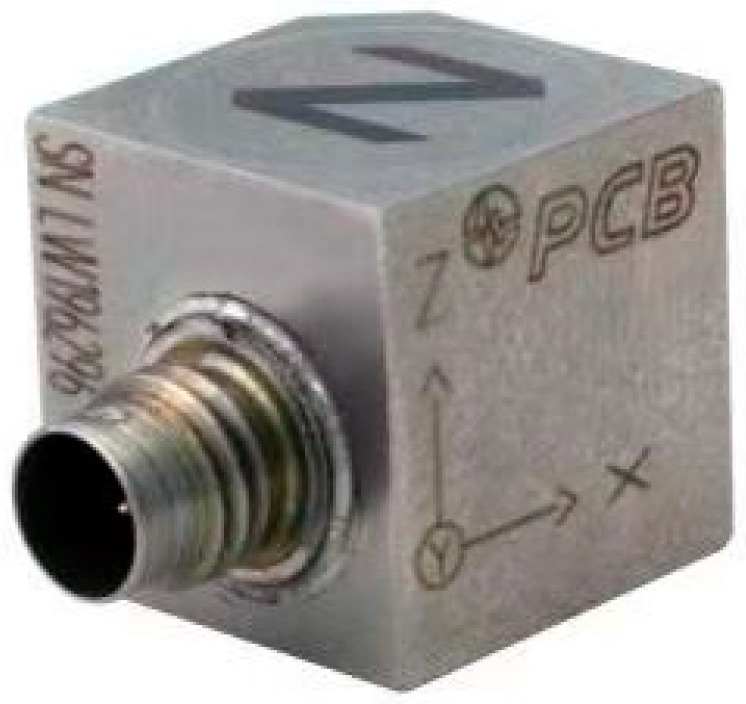
356A24 type triaxial accelerometer.

The 356A24 three-axis acceleration sensor, produced by PBC PIEZOTRONICS, USA, is suitable for high-precision engineering analysis, as shown in Figure 4. It can handle accelerations ranging from very small vibrations to intense dynamic motion. It can simultaneously measure acceleration along three mutually perpendicular axes (X, Y, Z), which is important for understanding and analyzing the behavior of objects in complex dynamic environments. Its specific parameters are shown in Table 2.

Lubricating oil was used as the test medium, Mobil 412 lubricating oil, with a viscosity of 14cSt at 100 °C and a flash point of 272 °C.

For comparison, the test materials used in this study were made of the same metals as those used in marine diesel engine cylinder liners and piston rings. The test medium was in the form of pins and disks. Their main parameters are as follows. The pin is a cylindrical material with a diameter of 3 mm and a length of 20 mm, made of gray cast iron, and mainly composed of metal elements such as carbon, iron, and manganese. The disk is a square disk material with a length of 40 mm, a width of 30 mm and a height of 3 mm, made of alloy cast iron, with a main composition similar to that of the pin, and quenched on the surface with good wear resistance.

### 3.4. Test Scheme

**Figure 5:**
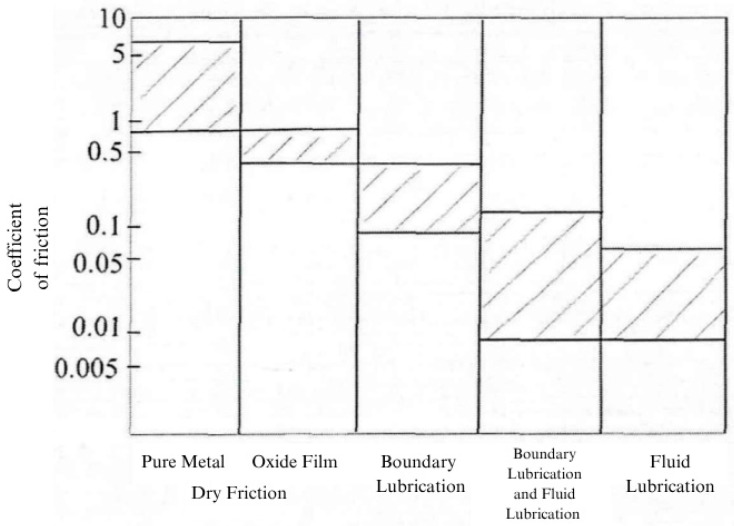
Lubrication status corresponding to the coefficient of friction.

The friction state can be indirectly altered by changing the lubrication conditions. In this study, three friction states—mixed, boundary, and dry—were adopted as the test conditions. As shown in Figure 5, these states can be identified from the friction coefficient, which illustrates the lubrication states corresponding to different friction coefficient values.

**Figure 6:**
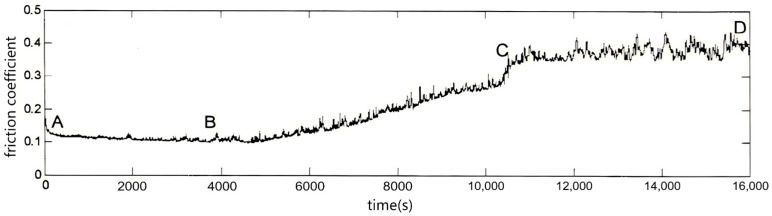
Experimental friction coefficient. AB: mixed friction; BC: boundary friction; CD: dry friction.

Three friction states—mixed, boundary, and dry—were achieved by changing the lubrication conditions during the test. To begin, an appropriate amount of lubricating oil was added to the oil box of the test bench to submerge the disk and start the mixed friction test. After 60 min of mixed friction, the test pin was kept running while all lubricating oil was drained from the oil box using a syringe. As the test continued, the lubricating oil on the friction pair surface gradually decreased, and the friction pair entered the boundary friction state. As no lubricating oil entered the test bench, the temperature gradually rose, the friction condition deteriorated, and the friction pair gradually entered the dry friction state. The friction coefficient collected during the test is shown in Figure 6. Based on the friction coefficient ranges corresponding to each lubrication state in Figure 5, section AB was identified as mixed friction, section BC as boundary friction, and section CD as dry friction. During the test, the load was fixed at 50 N, the reciprocating frequency was 0.5 Hz, and the stroke length was 8 mm. The friction vibration signal and friction coefficient were collected using the equipment [11,12].

The pin specimen and the square specimen were fixed on the friction and wear testing machine, ensuring that the pin specimen was suspended and did not touch the square specimen. At a selected frequency of 0.5 Hz and a reciprocating stroke of 8 mm, the m+p friction vibration signal acquisition system was used with a sampling frequency of 51,200 Hz. A noise signal lasting 2 s was collected, along with friction vibration signals under three different friction states. The collected data were then imported into MATLAB for processing using the CEEMD-based noise reduction algorithm. In addition, after denoising the original signal in the experiment, the extracted noise signal was compared with the laboratory noise using multi-scale permutation entropy to verify the denoising effect.

## 4. Results and Discussion

### 4.1. Algorithm Decomposition of Reciprocating Friction Simulation Signals

**Figure 7:**
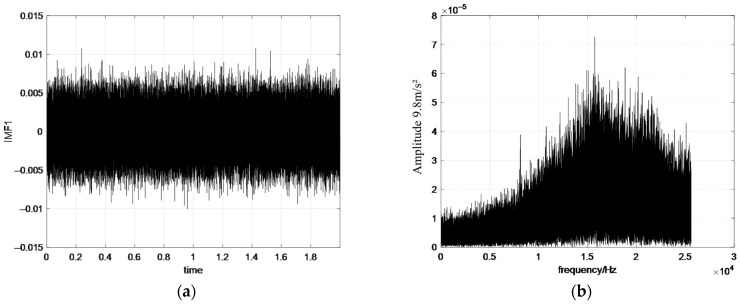

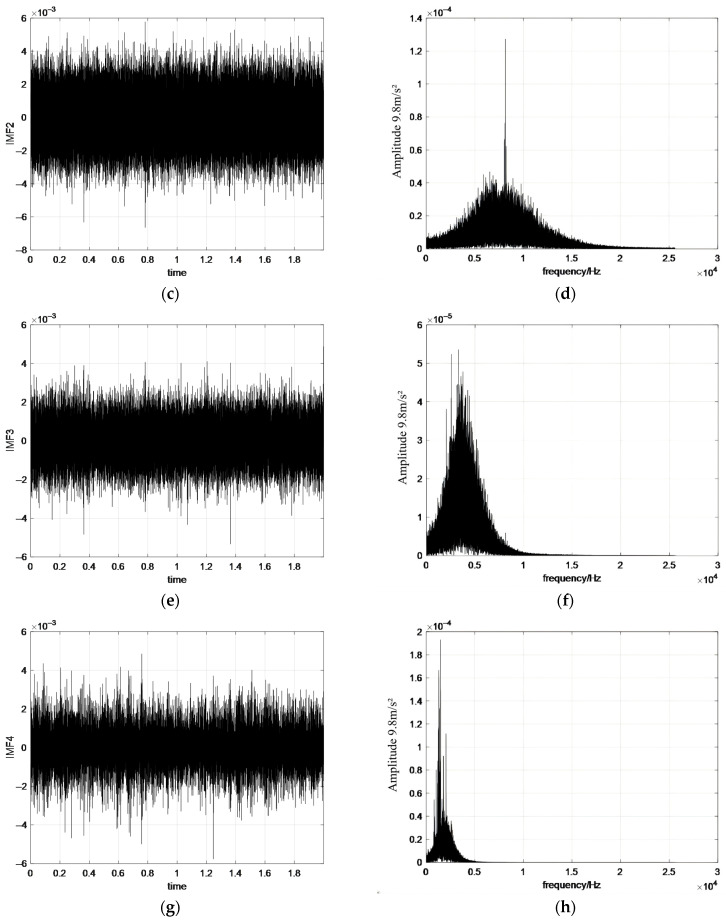
The IMF components obtained by processing the simulated signal with EEMD. (**a**) IMF1 waveform; (**b**) IMF1 spectrum; (**c**) IMF2 waveform; (**d**) IMF2 spectrum; (**e**) IMF3 waveform; (**f**) IMF3 spectrum; (**g**) IMF4 waveform; (**h**) IMF4 spectrum.

The synthesized simulation signal is imported into MATLAB, and the simulation signal is processed by the EEMD algorithm to obtain the corresponding IMF components as shown in Figure 7.

**Figure 8:**
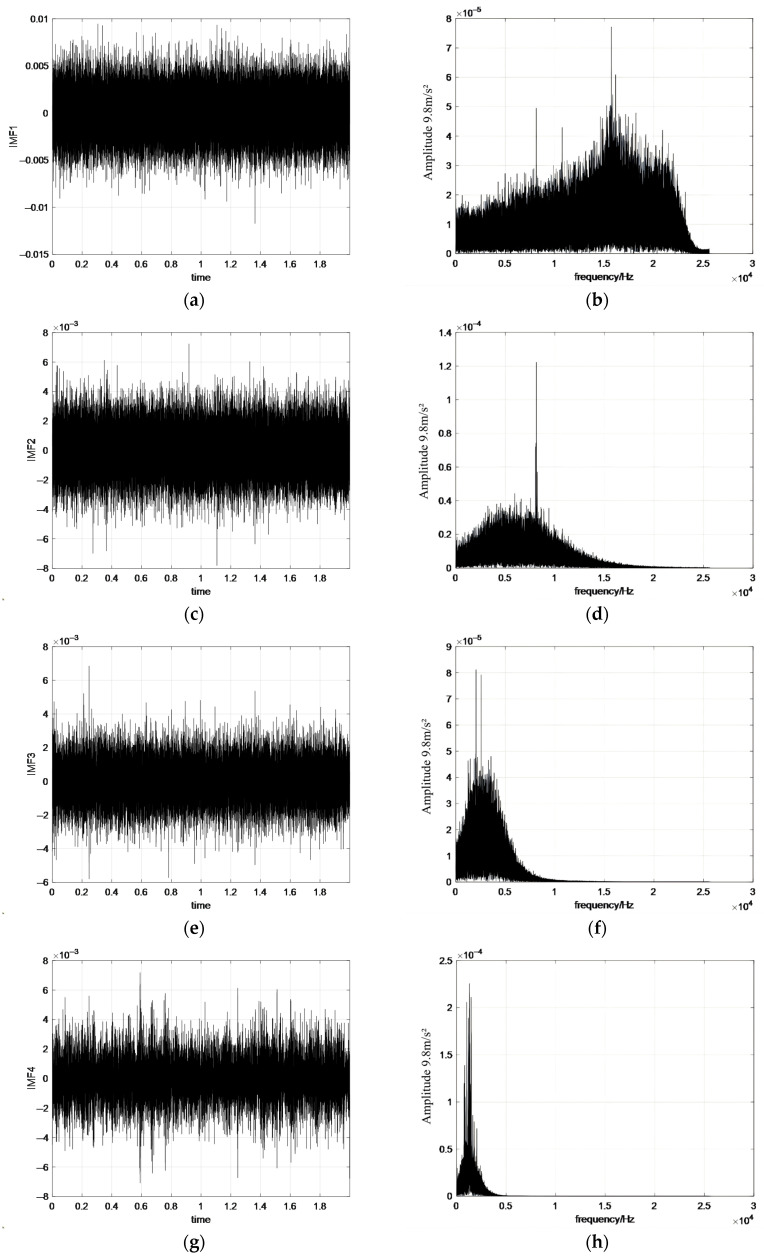
The IMF components obtained by processing the simulated signal with CEEMD. (**a**) IMF1 waveform; (**b**) IMF1 spectrum; (**c**) IMF2 waveform; (**d**) IMF2 spectrum; (**e**) IMF3 waveform; (**f**) IMF3 spectrum; (**g**) IMF4 waveform; (**h**) IMF4 spectrum.

The simulation signal is decomposed by the CEEMD algorithm to obtain the corresponding IMF components as shown in Figure 8.

As shown in Figure 7 and Figure 8, the frequencies of the IMF components gradually decrease as the decomposition order increases, with frequency bands arranged from high to low. In the EEMD, mode mixing can be observed when neighboring IMFs share similar frequency components, indicating interference during the decomposition process. In contrast, the IMFs obtained using CEEMD show clearer separation, especially in the lower-order components. Although the spectral characteristics of individual IMFs may appear qualitatively similar between the two methods, the improvement of CEEMD is reflected in its substantially reduced residual noise and enhanced reconstruction quality. These results indicate that CEEMD more effectively separates signal components from noise and suppresses inter-modal interference, thereby reducing mode mixing and improving decomposition accuracy.

**Figure 9:**
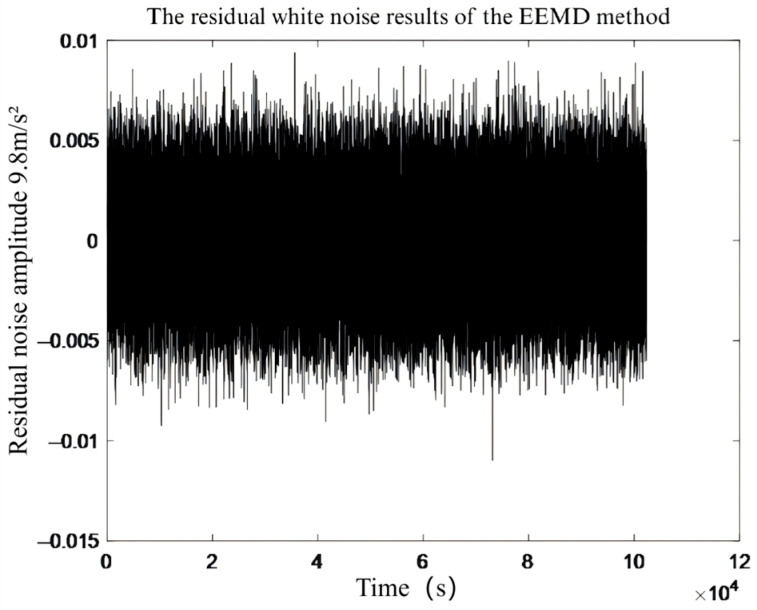
Residual white noise of EEMD method.

**Figure 10:**
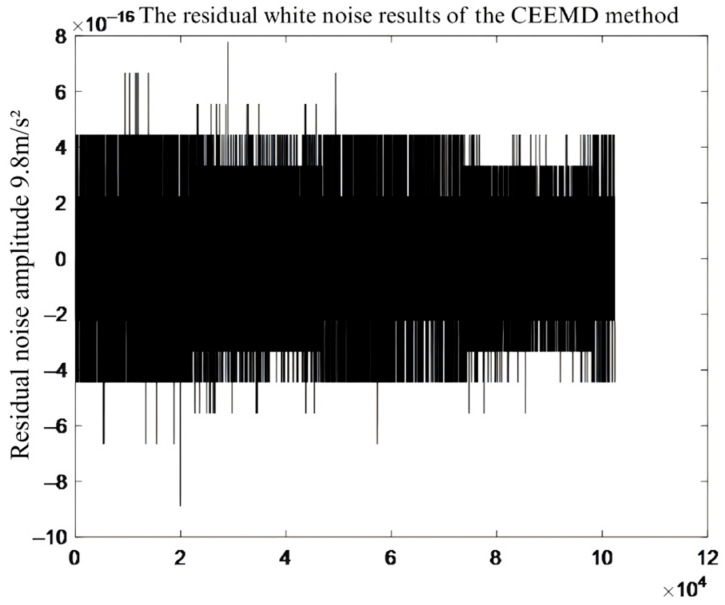
Residual white noise of CEEMD method.

To verify the effectiveness of the noise reduction effect of the EEMD and CEEMD algorithm, the noise signal collected from the laboratory was superimposed with the simulation signal obtained when the pin and block specimens were not in contact—to form a synthetic noisy signal. Both algorithms were then used to decompose this signal. As can be seen from Figure 9 and Figure 10, the residual noise from CEEMD is substantially smaller than that from EEMD, with a difference in several orders of magnitude. This indicates that CEEMD controls noise levels more precisely, reduces the error introduced by noise, and improves decomposition accuracy and resolution. In addition, in terms of the time consumption of the two algorithms, the EEMD algorithm took 913 s, while the CEEMD algorithm only required 131 s, indicating that the CEEMD algorithm has a higher decomposition efficiency, which helps to improve the efficiency of signal reconstruction.

### 4.2. IMF Component Screening

Three metrics are commonly used to evaluate filtering performance: signal-to-noise ratio (SNR), mean square error (MSE), and normalized cross-correlation (NCC). The SNR measures the ratio of signal strength to background noise. The MSE quantifies the difference between the estimated signal and the true signal. NCC indicates the similarity between two sequences, with values ranging from −1 to 1.

In this experiment, the processing effect of the denoising method was evaluated using the SNR, NCC, and MSE. Higher SNR and NCC values indicate better filtering performance, as they reflect a stronger signal relative to noise and greater similarity between the filtered and original signals. Conversely, a lower MSE value indicates better performance, since it represents a smaller error between the filtered signal and the original signal.

Three screening methods were compared: direct spectrum screening, kurtosis screening and adaptive correlation coefficient screening. Among them, the noise reduction method combining the CEEMD algorithm with adaptive correlation coefficient screening had the best effect on signal processing. The SNR reached 24.371 and the MSE was 9.611. NCC was 0.304, which confirmed the effectiveness of the noise reduction method used in this study.

The parameter values for each screening method using EEMD and CEEMD are summarized in Table 3 and Table 4. After processing the signal with CEEMD combined with the adaptive correlation coefficient screening method, the SNR improved significantly, the MSE decreased, and the NCC increased. These results confirm the effectiveness of the denoising method used in this study.

### 4.3. Validation of Multi-Scale Permutation Entropy

Permutation entropy is a method used to assess the complexity of time series by calculating the entropy based on the frequency of occurrence of each permutation pattern [13].

Permutation entropy offers several advantages: it is simple to calculate, robust to noise, and sensitive to nonlinear data. Multi-scale permutation entropy processes signals by constructing the “coarse-graining” of time series based on the permutation entropy algorithm [14].

To validate the general applicability of the proposed CEEMD-based denoising method, original signal segments obtained from the friction and wear testing machine were extracted at 2, 5, 10, 15, and 20 min under three distinct friction states—mixed, boundary, and dry—for CEEMD and reconstruction.

**Figure 11:**
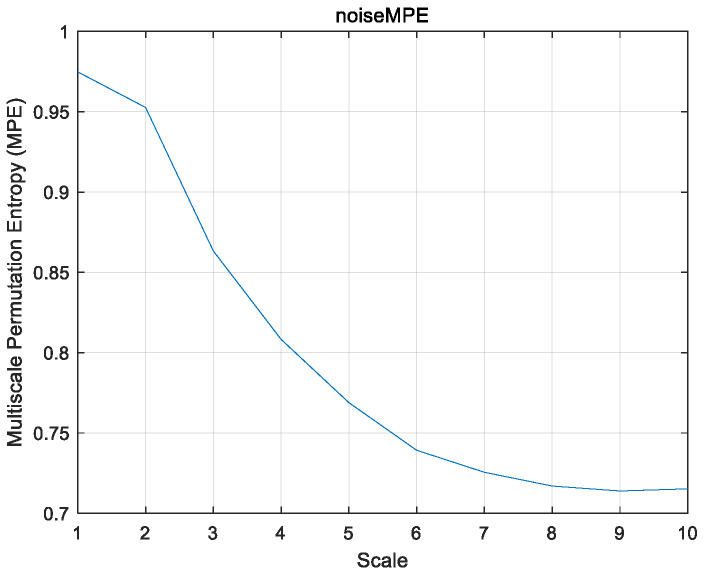
MPE curve of laboratory noise.

The multi-scale permutation entropy image of the laboratory noise is shown in Figure 11.

**Figure 12:**
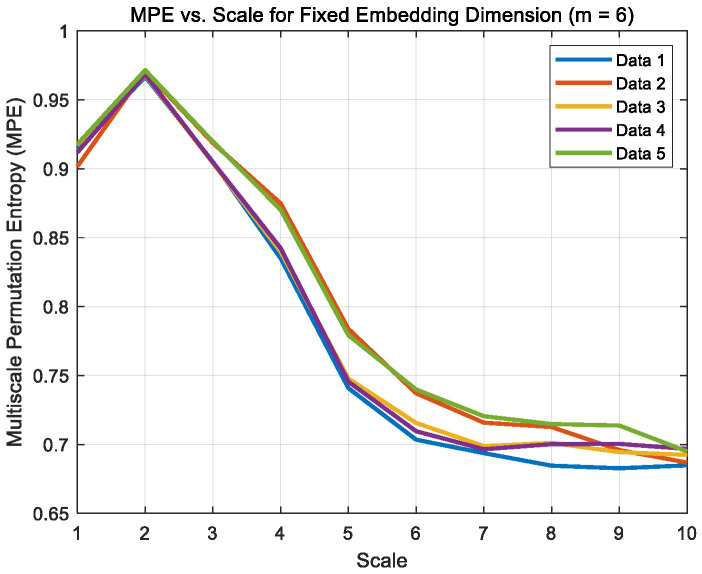
MPE curve of dry friction noise.

**Figure 13:**
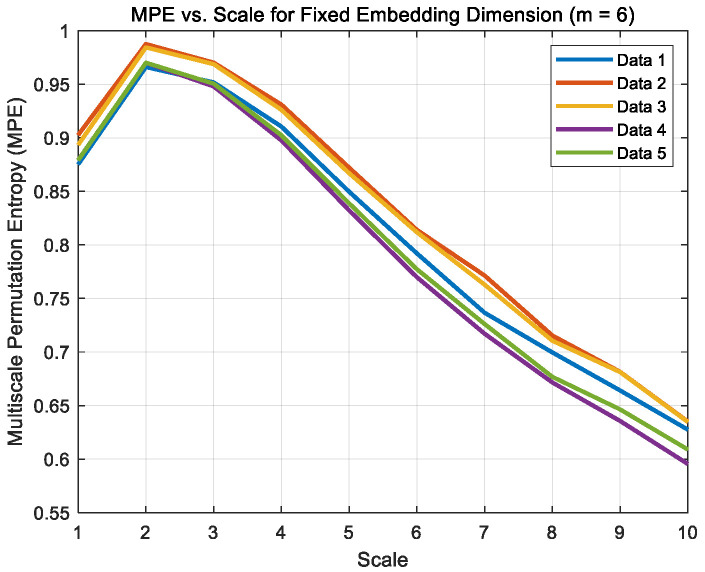
Boundary friction noise MPE curve.

**Figure 14:**
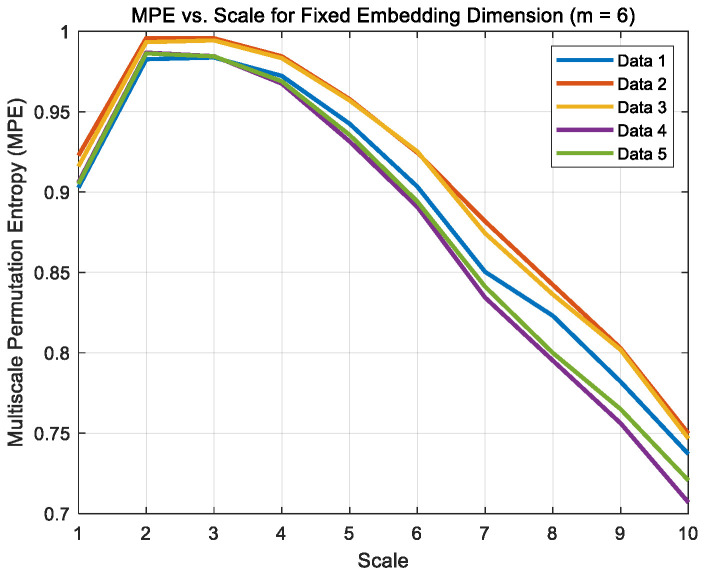
Mixed friction noise MPE curve.

Signals were captured at time points 2, 5, 10, 15, and 20 min under dry, boundary and mixed friction states, denoted as Data1–Data5 for each condition, respectively. The algorithm parameters were set in MATLAB with an embedding dimension of 6, a time delay of 1, and a scale factor of 10. Their multi-scale permutation entropy images are shown in Figure 12, Figure 13 and Figure 14.

The multi-scale permutation entropy curves of the denoised noise signals under the three different states were compared with those of the laboratory noise signal using software, and the correlation coefficients were calculated. The results are presented in Table 5. 

The results show that under dry, boundary, and mixed friction conditions, the multi-scale permutation entropy curves of the signals processed by CEEMD are highly consistent with those of the laboratory noise signals, with correlation coefficients of 0.803, 0.812, and 0.869, respectively. Although the correlation coefficient for dry friction is only slightly above 0.8, this marginal difference can be attributed to the physical characteristics of dry friction. Under dry friction conditions, the absence of a lubricating film leads to stronger impact and higher randomness in the vibration signal, making complete noise extraction more challenging. Nevertheless, the coefficient remains above 0.8, indicating a high degree of similarity.

The high correlation between the denoised signals and the laboratory noise is physically significant. In the experimental setup, the laboratory noise represents the baseline vibration environment when no friction contact occurs. When a denoised signal correlates strongly with this baseline, it indicates that the proposed CEEMD-based method effectively removes friction-induced vibration components while preserving the underlying noise characteristics of the test environment. This suggests that the denoising process successfully separates the friction signal from background noise without introducing artifacts, thereby enabling more accurate analysis of friction-induced vibrations. The consistently high correlation across all three friction states further validates the robustness and effectiveness of the proposed denoising method.

**Figure 15:**
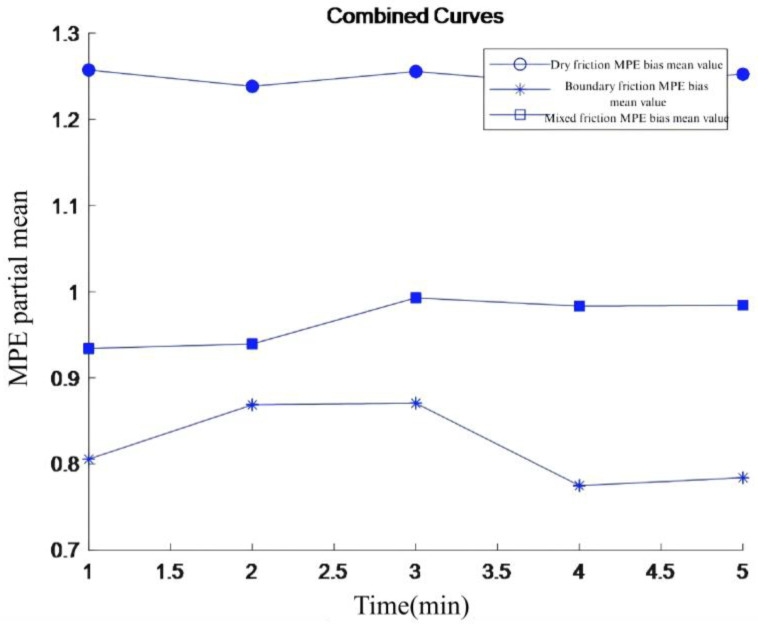
Mean bias of MPE under three friction states.

The multi-scale permutation entropy of the denoised signal was first calculated using the method described in the previous section. The partial mean was then computed from the multi-scale permutation entropy values at these five time points to obtain the curve shown in Figure 15.

After denoising, the multi-scale permutation entropy mean values of vibration signals under different friction states show different curve characteristics. By comparing the similarity between the multi-scale permutation entropy mean value curves of vibration signals under the unknown state and the known state, the unknown state can be classified. This method is expected to improve the accuracy of friction state identification, thereby enhancing efficiency.

It should be noted, however, that the above results are based on a single set of trials, with five time points (2, 5, 10, 15, and 20 min) selected under each friction state to capture temporal evolution rather than repeated independent experiments. All measurements were performed using the same calibrated instruments (BRUKER UMT tribometer and 356A24 accelerometer), ensuring consistent instrumentation errors across all trials. While repeated trials and statistical uncertainty quantification would further strengthen the validation, the observed differences—such as the 16-order-of-magnitude reduction in residual noise and the consistently high correlation coefficients (>0.8)—are substantially larger than any possible instrumentation or random variability. This provides strong support for the effectiveness of the proposed method. In future work, we will conduct repeated experiments and report measurement variability.

## 5. Conclusions

In this study, the original simulation signal was first synthesized by combining the simulation signal with the laboratory noise signal. After comparing the “EMD”-like methods and different methods for screening modal components, the correlation coefficient adaptive screening method was selected to screen appropriate components and perform reconstruction. The signal reconstruction was verified through the evaluation index of filtering effect, and then the accuracy and effectiveness of the denoised signal were verified through the multi-scale permutation entropy algorithm of the noise signal, achieving effective denoising. The following conclusions were drawn through analysis:

(1) After decompressing the simulated vibration signal and reconstructing it using the CEEMD-based adaptive correlation coefficient screening method, the noise reduction effect was evaluated by using indicators such as the SNR, MSE and NCC. For the reconstructed simulated signal using the adaptive correlation coefficient screening method, the SNR was 24.371, the MSE value was 9.611 and the NCC value was 0.304, indicating that this method has advantages in reconstructing friction vibration signals. Compared with other advanced methods such as wavelet thresholding, CEEMDAN, and VMD, the proposed method offers an effective combination of adaptive decomposition, noise suppression capability, and computational efficiency, demonstrating its potential for practical applications in marine engineering.

(2) After noise reduction using the CEEMD adaptive correlation coefficient screening method, some deviation remains between the friction vibration signal and the noise under different states; however, the overall correlation coefficients are above 0.8, indicating a high degree of correlation. The results show that the noise signals obtained by the noise reduction method in this study are highly correlated with the laboratory noise, indirectly confirming the accuracy of the denoising process. Meanwhile, introducing the multi-scale permutation entropy bias mean as a characteristic parameter can effectively support the subsequent differentiation of different friction states and improve the resolution efficiency.

(3) We acknowledge that the core denoising algorithm (CEEMD) used in this study is a well-established technique. However, the novelty of this work lies not in the algorithm itself, but in its systematic adaptation and validation for the specific challenges of reciprocating friction vibration signals under marine engine conditions. Due to the limitations of experimental conditions and time, the CEEMD-based noise reduction and analysis method for reciprocating friction vibration signals proposed in this study still needs further improvement. In future work, we plan to extend the experimental conditions by adjusting the lubricant and increasing the load to improve the authenticity of the simulation environment. We will further investigate the directionality of vibration and its influence on the running-in process. In addition, we intend to explore the composition and effects of background noise sources from equipment and environment to improve the robustness of the denoising method. Moreover, we will employ machine learning algorithms using the multi-scale permutation entropy partial as an input feature to accelerate friction state recognition and improve identification efficiency.

## Figures and Tables

**Table 1:** Key parameters of the CEEMD algorithm used in this study.

Parameter	Value	Justification
Ensemble number I	8	Paired positive and negative white noise; 8 trials in CEEMD are equivalent to 100 trials in EEMD
Noise amplitude coefficient *ε_k_*	0.2	Ratio of added noise standard deviation to signal standard deviation
Stopping criterion for IMF extraction	IMF conditions + residual condition	(a) Extrema and zero-crossings differ by at most one; (b) mean of envelopes is zero; decomposition stops when residual has ≤2 extrema
Sampling frequency	51,200 Hz	Consistent with experimental data acquisition
Signal duration	2 s	Length of each analyzed signal segment

**Table 2:** Specifications of the model 356A24 triaxial accelerometer.

Axial Direction	X-Axis	Y-Axis	Z-Axis
Sensitivity	9.89 (mV/g)	10.9 (mV/g)	9.96 (mV/g)
Frequency response (Hz)	0.5~12,000	0.5~12,000	0.5~12,000
Measurement range (g pk)	±500	±500	±500
Broadband resolution (g rms)	0.002	0.002	0.002

**Table 3:** Parameter values for each screening method of EEMD.

Screening Methods	SNR	MSE	NCC
Spectrum direct filtering	0.019	10.166	0.004
Kurtosis screening	6.415	10.317	−0.0433
Adaptive screening of correlation coefficients	6.435	10.318	−0.0163

**Table 4:** Parameter values for each screening method in CEEMD.

Screening Methods	SNR	MSE	NCC
Spectrum direct filtering	0.043	10.167	0.005
Kurtosis screening	6.350	10.018	0.122
Adaptive screening of correlation coefficients	24.371	9.611	0.304

**Table 5:** Correlation coefficients of noise signal MPE with laboratory noise MPE curves under each friction state.

Friction State	Correlation Coefficient	Degree of Correlation
Dry friction	0.803	High
Boundary friction	0.812	High
Mixed friction	0.869	High

## Data Availability

The original contributions presented in this study are included in the article. Further inquiries can be directed to the corresponding author.
